# Surgical strategies and sequencing decisions for ankylosing spondylitis with severe hip deformity

**DOI:** 10.1016/j.jor.2026.05.007

**Published:** 2026-05-08

**Authors:** Zihe Niu, Shihao Hong, Xin Yang

**Affiliations:** Department of Orthopedics, Peking University First Hospital, Beijing, 100034, China

**Keywords:** Ankylosing spondylitis, Spinal osteotomy, Total hip arthroplasty, Spinopelvic parameters

## Abstract

**Background:**

Advanced ankylosing spondylitis (AS) may involve both the thoracolumbar spine and the hips, resulting in a complex deformity characterized by kyphosis, pelvic retroversion, and hip ankylosis. In severe cases, both pedicle subtraction osteotomy (PSO) and total hip arthroplasty (THA) may be required, but the optimal surgical sequence remains controversial.

**Main body:**

This controversy arises from the biomechanical coupling of the spine–pelvis–hip complex. Spinal correction can restore sagittal balance and alter pelvic orientation, thereby affecting the functional position of the acetabular component in THA. Conversely, severe hip ankylosis and contracture may compromise patient positioning, acetabular exposure, and even the feasibility of spinal correction. This review summarizes the biomechanical rationale, clinical indications, advantages, and limitations of spine-first and hip-first strategies, and highlights that surgical sequencing should be determined according to deformity dominance, surgical feasibility, and the anticipated postoperative spinopelvic environment.

**Conclusions:**

Neither spine-first nor hip-first surgery is universally appropriate for patients with AS and severe hip deformity. Surgical sequencing should be individualized through comprehensive assessment of spinal deformity, pelvic compensation, hip pathology, and expected changes in spinopelvic alignment after reconstruction.

## 1 Introduction

Ankylosing spondylitis is a spondyloarthropathy characterized by chronic inflammation of the axial skeleton, osteophyte formation, and progressive ankylosis. In its advanced stage, it may lead not only to thoracolumbar kyphosis but also frequently involve the hip joints, resulting in pain, restricted motion, and even bony ankylosis^1^, ^2^, ^3^. The hip joint is the most commonly affected peripheral joint, with involvement reported in 30% to 50% of patients with ankylosing spondylitis; among these patients, 47% to 90% present with bilateral involvement.^4^^,^^5^ Hip involvement can have a substantial impact on the overall quality of life of patients with ankylosing spondylitis; therefore, timely and effective treatment is of critical importance.

With the development of biologic therapies and standardized rehabilitation programs, disease progression can be delayed in some patients. However, for those who have already developed end-stage hip involvement or severe thoracolumbar kyphosis, surgery remains a key intervention for restoring posture, improving function, and enhancing quality of life^6^, ^7^, ^8^. In complex cases, spinal correction and hip reconstruction are often not two independent issues. Thoracolumbar kyphosis can shift the center of gravity anteriorly and induce compensatory posterior pelvic tilt, whereas hip ankylosis and flexion contracture may further aggravate the flexed posture of the trunk, resulting in the so-called “folding man” deformity.^9^^,^^10^ At this stage, both pedicle subtraction osteotomy (PSO) and total hip arthroplasty (THA) may become essential components of the reconstructive strategy. However, whether the spine or the hip should be addressed first remains a matter of ongoing debate, with no universal consensus to date. The source of this controversy is not merely a matter of academic preference. Rather, spinal correction can substantially alter pelvic orientation and the functional position of the acetabular cup, whereas severe hip ankylosis may directly affect anesthetic management, prone positioning, surgical exposure, and identification of the true acetabulum.

Accordingly, this review approaches the issue from an integrated “spine–pelvis–hip” perspective and focuses on the central question of surgical sequencing. By examining operative strategies for ankylosing spondylitis complicated by severe hip deformity, we aim to provide a useful reference for clinical decision-making regarding the optimal order of intervention.

## 2 Key dimensions of preoperative assessment

### 2.1 Spinal alignment and pelvic compensation

Qian et al. found that, as kyphotic spinal deformity progresses in patients with ankylosing spondylitis, the center of mass of the trunk shifts anteriorly and the sagittal vertical axis (SVA) increases. To maintain sagittal balance, the pelvis undergoes compensatory retroversion, manifested by an increase in pelvic tilt (PT) and a decrease in sacral slope (SS).^11^ Lin and colleagues found that an increase in pelvic tilt (PT) is the most important compensatory mechanism by which patients with ankylosing spondylitis maintain standing balance.^12^ Qian even observed negative values of sacral slope (SS), indicating that pelvic compensation had reached its anatomical limit and that the hip joint was in a state of extreme hyperextension.^11^ However, this compensation is not a unidirectional process, but rather a bidirectional coupling among the spine, pelvis, and hip. A recent review pointed out that, as the intermediary structure linking the spine and the hip, the pelvis not only influences sagittal balance but also determines the functional orientation of the acetabulum.^13^ Therefore, preoperative planning for THA should not rely solely on static imaging or the traditional “safe zone” to determine cup position, but should also take functional changes under different postural conditions into careful consideration.

In patients with ankylosing spondylitis, hip involvement itself may in turn affect the compensatory capacity of the pelvis. In a study of 182 patients with ankylosing spondylitis, Guo and colleagues found that, as radiographic hip involvement became more severe, both the pattern of pelvic tilt and the mode of lower-limb compensation changed accordingly. Patients with severe hip involvement exhibited lower PT, higher SS, and greater SVA, suggesting that advanced hip disease may impair the ability of the pelvis to tilt posteriorly in order to compensate for spinal deformity.^14^ This finding suggests that, in patients with ankylosing spondylitis complicated by end-stage hip damage, the compensatory chain of “spinal kyphosis–pelvic retroversion” may be disrupted by hip ankylosis or flexion contracture, thereby affecting the overall preoperative assessment and the choice of surgical sequence.

In addition, pelvic compensation is clearly posture-dependent. In an EOS-based study of patients with thoracolumbar kyphosis secondary to ankylosing spondylitis, Zhao and colleagues showed that changes in pelvic orientation from standing to sitting were closely associated with overall spinal alignment.^15^ In other words, the pelvis is not a static “base,” but a dynamic structure whose orientation changes with the transition between standing and sitting, as well as before and after spinal correction. Therefore, preoperative assessment in patients with ankylosing spondylitis complicated by severe hip deformity should not be limited to a single static parameter. Instead, spinal alignment, pelvic orientation, and hip morphology/range of motion should be evaluated as parts of an integrated functional system.

### 2.2 Functional imaging and dynamic assessment

The central biomechanical basis underlying the controversy over surgical sequencing is that pelvic rotation can directly alter the functional inclination and anteversion of the acetabular cup. In a study of patients with ankylosing spondylitis undergoing primary THA, Gu and colleagues showed that the sagittal pelvic parameters followed the fundamental relationship of Pelvic Incidence(PI)= PT + SS, and that both functional cup inclination and functional anteversion were positively correlated with PT and negatively correlated with SS(16). This means that the more the pelvis tilts posteriorly, the greater the functional anteversion and inclination of the acetabular cup. On this basis, the authors proposed that, in patients with ankylosing spondylitis, systematic evaluation of sagittal parameters such as PI, PT, and SS is essential before THA, given the presence of spinal flexion deformity and compensatory pelvic retroversion, in order to reduce the risk of postoperative impingement, wear, and dislocation.

This conclusion has been more directly validated in studies on spinal correction. Using CT-based measurements, Hu and colleagues found that both acetabular inclination and anteversion decreased significantly after lumbar PSO in patients with thoracolumbar kyphosis secondary to ankylosing spondylitis. Moreover, these changes were significantly correlated with the change in PT, suggesting that spinal correction not only restores kyphotic deformity and pelvic retroversion but also helps return abnormal acetabular orientation toward a relatively normal state.^16^ Zhao and colleagues further confirmed, in patients who had undergone THA followed by PSO, that acetabular anteversion decreased significantly after PSO, and that the magnitude of this change was associated with the degree of spinal deformity correction. This finding suggests that once the spine is corrected after THA, the functional cup position originally established on the basis of the preoperative compensatory pelvic state may be fundamentally altered.^17^

Therefore, in patients with ankylosing spondylitis, the safe zone for cup placement should be understood as a functional safe zone rather than a fixed anatomic one. A systematic review by Sudhan and colleagues likewise noted that the spine–pelvis–hip complex undergoes proportional postural changes across different positions, and that the traditional Lewinnek safe zone cannot adequately account for all patients with spinal stiffness, sagittal imbalance, or abnormal pelvic mobility. In patients with ankylosing spondylitis complicated by severe spinal kyphosis or hip ankylosis, if pelvic retroversion and its reversible changes after spinal correction are overlooked, and the acetabular cup is placed solely according to conventional empirical angles, postoperative problems such as anterior impingement, posterior dislocation, or restricted range of motion may occur.^13^

### 2.3 Hip structural damage and range of motion

From the perspective of surgical sequencing, the truly critical issue in patients with the so-called “folding man” deformity is not simply which procedure—spinal correction or hip surgery—is more convenient to perform first. Rather, the sequence itself may alter the final pelvic orientation, the arc of pelvic motion during postural transitions, and the functional environment of the acetabular cup. Zhao's study showed that, in patients with ankylosing spondylitis, the change in the pelvic plane angle from standing to sitting was pronounced before PSO, whereas this change was significantly reduced after PSO, indicating that spinal correction can reshape the pattern of pelvic motion during everyday postural transitions.^15^ More importantly, these changes were closely associated with parameters such as SVA and overall spinal alignment, suggesting that pelvic mobility is not an isolated hip-related phenomenon, but rather a downstream manifestation of global sagittal alignment.

Li and colleagues further provided more direct evidence from the perspective of surgical sequencing. Their study found that the lumbar sagittal profile in patients with ankylosing spondylitis–related thoracolumbar kyphosis significantly affects pelvic orientation and pelvic motion during the transition from standing to sitting. On this basis, the authors proposed that if THA is performed before PSO, patients with different lumbar sagittal patterns may face different directions of prosthetic dislocation risk, whereas a PSO-first strategy may be more conducive to THA planning within a pelvic environment closer to its final postoperative state.^18^ Zhao and colleagues also supported this view in their study of patients who underwent PSO after THA. They found that the decrease in acetabular anteversion after PSO was correlated with the magnitude of spinal correction. This suggests that, in patients who undergo THA first, additional consideration must be given to the future pelvic rotation induced by subsequent spinal correction; otherwise, the cup position may fall outside the originally anticipated functional safe range after the second-stage spinal procedure.^17^

On the other hand, Hu's study showed that PSO can restore abnormal acetabular orientation toward a more normal alignment.^16^ This provides biomechanical support for a spine-first strategy: when compensatory pelvic retroversion is pronounced and substantial changes in PT and SS are anticipated after spinal correction, it is more advantageous to first reconstruct the upstream spine–pelvis relationship and then proceed with THA, so that cup position can be determined on the basis of the final pelvic posture. Conversely, if the patient has severe hip flexion contracture or bony ankylosis that already markedly limits positioning and the feasibility of spinal correction, the hip may still need to be addressed first in clinical practice, even though a spine-first approach is theoretically more consistent with the logic of cup-position planning. Taken together, the essence of the sequencing controversy lies in the balance between the stability of functional cup positioning and the immediate feasibility of surgery.

### 2.4 Surgical feasibility and exposure

Based on the biomechanical relationships described above, preoperative assessment in patients with ankylosing spondylitis complicated by severe hip deformity should cover at least four dimensions. First, the degree of sagittal spinal imbalance should be evaluated, including SVA, PT, SS, PI, and overall standing posture, in order to determine whether the pelvis is in a state of marked compensatory retroversion and whether substantial recoil may occur after subsequent spinal correction^11^^,^^15^^,^^16^^,^^18^^,^^19^. Second, functional-position imaging should be used whenever possible, including standing and sitting whole-spine images as well as pelvis-related imaging, rather than relying solely on a single supine view or standard anteroposterior pelvic radiographs. Only by incorporating postural variation into preoperative planning can the functional cup position be assessed more accurately.^13^ Third, the severity of hip involvement itself must be incorporated into the spinopelvic assessment rather than being treated merely as an “associated lesion.” Guo and colleagues pointed out that patients with severe hip involvement have a reduced capacity for compensatory pelvic retroversion. Therefore, in patients with ankylosing spondylitis awaiting THA, the extent of hip involvement and its impact on sagittal balance should be specifically evaluated during preoperative counseling and surgical planning.^14^ Fourth, attention should also be paid to the structural damage of the hip joint and preoperative range of motion. Man and colleagues reported in patients with ankylosing spondylitis undergoing THA that greater severity of femoral head erosion was associated with a longer time to postoperative recovery of independent ambulation without walking aids, while preoperative Range of motion (ROM) and the degree of joint space narrowing also influenced postoperative improvement in mobility.^20^ This suggests that, when deciding whether to address the spine or the hip first, one should not only analyze how changes in pelvic parameters may affect cup positioning, but also evaluate the difficulty of true acetabular exposure, the degree of bony fusion, soft-tissue contracture, and structural changes in the proximal femur, as these factors directly determine whether THA itself can be performed safely.

In summary, surgical sequencing in patients with ankylosing spondylitis complicated by severe hip deformity should not be based solely on the severity of a single local lesion. Rather, it should be grounded in an integrated assessment encompassing spinal alignment, pelvic orientation, and hip structure and mobility. Only when the preoperative evaluation can simultaneously answer the following three questions—how the pelvis is likely to rotate in the future, what functional environment the acetabular cup will ultimately face, and whether surgery is technically feasible at the current stage—can the choice of surgical sequence be considered truly biomechanically sound.

## 3 Spine-first strategy

From the perspective of the integrated biomechanics of the spine–pelvis–hip complex, the theoretical rationale for a spine-first strategy is to first restore upstream sagittal balance and pelvic orientation, and then perform THA planning and cup positioning within a pelvic environment that is closer to its final postoperative state^15^, ^16^, ^17^, ^18^. This strategy is particularly applicable to patients in whom thoracolumbar kyphosis predominates in the overall deformity, compensatory pelvic retroversion is pronounced, and substantial changes in PT, SS, and pelvic rotation are anticipated after spinal correction.

### 3.1 Rationale

As discussed above, pelvic retroversion in patients with ankylosing spondylitis increases the functional anteversion and inclination of the acetabular cup, whereas pelvic recoil after spinal correction may alter this abnormal functional orientation once again^16^, ^17^, ^18^, ^19^. Hu and colleagues confirmed that both acetabular inclination and anteversion decreased significantly after PSO, and that these changes were significantly correlated with changes in PT, suggesting that spinal correction not only improves forward trunk inclination but also restores acetabular orientation toward a relatively normal state.^16^ In other words, if a patient undergoes THA first in the presence of marked pelvic retroversion and subsequently receives PSO, the functional cup position originally established on the basis of the deformity state is likely to shift after spinal correction, thereby increasing the risks of impingement and instability.

This view was further reinforced by Zhao and colleagues in their study of patients who underwent PSO after THA. They found that acetabular anteversion decreased significantly from 18.59° to 5.85° after PSO, and that the magnitude of this change was associated with the degree of spinal deformity correction.^17^ This indicates that, in patients with pronounced preoperative pelvic compensation, if THA is performed before PSO, the functional environment of the cup is not fixed after surgery, but may be redefined by subsequent spinal correction. Therefore, from the perspective of long-term cup stability, a spine-first strategy is more consistent with the logic of first determining the final orientation of the pelvis and then proceeding with acetabular reconstruction.

### 3.2 Indications

A spine-first strategy is not universally applicable, but it is often theoretically more appropriate in the following situations. First, the patient's overall deformity is predominantly driven by thoracolumbar kyphosis and sagittal imbalance, with a markedly increased standing SVA, pronounced pelvic retroversion, and preoperative assessment suggesting that pelvic orientation will recoil substantially after PSO(11, 12, 15, 17, 19). Second, although hip involvement is present, the hip flexion contracture is not yet severe enough to compromise anesthetic management or the positioning required for spinal surgery^10^^,^^21^, ^22^, ^23^. Third, subsequent THA is expected to be performed under relatively controllable exposure conditions; in this setting, prioritizing restoration of the spine–pelvis relationship is more conducive to reducing errors in cup-position planning.

Li and colleagues reported that the lumbar sagittal profile in patients with ankylosing spondylitis–related thoracolumbar kyphosis significantly affects pelvic orientation and pelvic motion during the transition from standing to sitting, and that patients with different lumbar curvature patterns may face different risks of dislocation if PSO is performed after THA(19). On this basis, the authors proposed that, in patients requiring both PSO and THA, performing PSO before THA is more favorable for hip reconstruction within a more stable pelvic environment. This finding provides relatively direct evidence supporting a spine-first sequencing strategy.

### 3.3 Advantages

The greatest potential advantage of a spine-first strategy lies in allowing subsequent THA to be planned and positioned according to the patient's final posture, rather than the preoperative compensatory posture. Zhao and colleagues found that, in patients with ankylosing spondylitis, pelvic orientation changed markedly from standing to sitting before PSO, whereas the magnitude of this change was significantly reduced after PSO, suggesting that spinal correction can reshape the pattern of pelvic motion during postural transitions.^15^ This indicates that a spine-first strategy affects not only static parameters such as PT and SS, but also the dynamic arc of pelvic motion during sitting and standing, thereby altering the true functional environment of the acetabular cup in daily life.

In this sense, the value of a spine-first approach lies not merely in “straightening the back” first, but more importantly in releasing the pelvis from its abnormal compensatory state before acetabular cup placement is performed on the basis of the newly established spine–pelvis relationship. For patients with marked pelvic retroversion and substantial anticipated changes in functional posture after surgery, this strategy may help reduce the risk of anterior impingement or posterior dislocation caused by excessive cup anteversion followed by pelvic anteversion after correction, and may also facilitate restoration of lower-limb length and reconstruction of the hip center in a manner more consistent with the demands of the final postural state^15^, ^16^, ^17^, ^18^.

### 3.4 Limitations and technical considerations

Although a spine-first strategy offers clear biomechanical advantages, its implementation presupposes that the patient can safely proceed to spinal surgery. In patients with severe bony hip ankylosis, marked flexion contracture, or even a “folding man” posture, anesthesia, turning, prone positioning, and intraoperative pressure injury prevention are themselves major challenges^21^, ^22^, ^23^, ^24^. Under such circumstances, even if a spine-first approach is more rational from the standpoint of final cup stability, it may still have to be abandoned in clinical practice because it cannot be performed safely at the current stage.

In addition, Guo and colleagues reported that, as radiographic hip involvement becomes more severe, the compensatory capacity for pelvic retroversion declines, as reflected by reduced PT, increased SVA, and worsening overall balance.^14^ This finding suggests that, in some patients with advanced hip disease, the hip is no longer merely a “downstream lesion,” but may instead become a key factor limiting pelvic adjustment and the feasibility of spinal correction. In other words, the theoretical applicability of a spine-first strategy decreases as hip ankylosis and flexion contracture become more severe.

### 3.5 Summary

In summary, a spine-first strategy is more suitable for patients in whom spinal deformity predominates, pelvic retroversion is pronounced, substantial changes in pelvic orientation and functional cup position are anticipated after PSO, and the positioning required for spinal surgery can be safely achieved. The principal advantage of this approach lies in restoring the upstream spine–pelvis relationship first, thereby reducing errors in subsequent THA cup positioning and the associated risk of instability. However, in patients whose hip deformity has already severely compromised positioning and perioperative safety, rigid adherence to the “biomechanically optimal sequence” is not realistic. In such cases, surgical sequencing must ultimately return to a more fundamental question: whether the procedure can be safely performed at the current stage.

## 4 Hip-first strategy

Unlike the spine-first strategy, which emphasizes restoration of the final pelvic environment before hip reconstruction, a hip-first approach is more often a pragmatic pathway driven by surgical feasibility. In patients with severe bony hip ankylosis, marked flexion contracture, difficulty turning, or an inability to safely achieve the positioning required for spinal surgery, performing THA first—or initially using hip surgery to release part of the contracture—is often a prerequisite for all subsequent reconstructive procedures.

### 4.1 Rationale

In patients with ankylosing spondylitis complicated by severe hip deformity, hip involvement is not limited to local pain and restricted motion. By constraining pelvic rotation and lower-limb positioning, it may in turn aggravate global sagittal imbalance. Guo and colleagues found that the more severe the hip involvement, the poorer the functional scores, the worse the overall sagittal balance, and the more altered the pattern of pelvic compensation.^14^ This suggests that, in some patients, hip ankylosis and flexion contracture have already become major drivers of imbalance within the entire spine–pelvis–hip complex. In such cases, if hip contracture and restricted motion are not addressed first, the positioning, turning, and execution of subsequent spinal surgery may be fundamentally limited.

Therefore, the theoretical basis of a hip-first strategy is not that it is more advantageous than a spine-first approach for cup-position planning, but rather that it addresses the factor currently posing the greatest obstacle to surgical feasibility. In patients who cannot be placed safely in the prone position, who have severely restricted lower-limb positioning, or whose pelvis is fixed in an extreme flexed posture, performing THA first, or undertaking related hip release/osteotomy procedures, may improve sitting, recumbency, turning, and standing conditions, thereby creating the operative conditions necessary for subsequent spinal correction^25^, ^26^, ^27^, ^28^.

### 4.2 Indications

A hip-first strategy is most appropriate for several groups of patients. First, it is particularly suitable for those in whom bony hip fusion, severe flexion contracture, or fixed bilateral hip deformity has already markedly interfered with anesthesia and the positioning required for spinal surgery^10^^,^^29^, ^30^, ^31^, ^32^, ^33^, ^34^. Second, it may be favored when the patient's principal symptoms arise from hip pain, limitations in sitting or lying, and impaired ambulation, whereas thoracolumbar kyphosis, although present, has not yet become the most urgent surgical bottleneck.^28^^,^^35^ Third, it is indicated when preoperative assessment suggests that spinal correction itself cannot be performed safely unless the hip-related constraints are addressed first.^21^

In addition, Man and colleagues reported that the severity of structural hip damage in patients with ankylosing spondylitis influences postoperative recovery after THA. Patients with more severe femoral head erosion required a longer time to regain independent ambulation without walking aids, and preoperative ROM, joint space narrowing, and the extent of structural destruction were also closely associated with postoperative recovery.^20^ These findings suggest that the severity of hip disease affects not only the patient's current function, but also the potential benefit and recovery pattern after THA itself. Therefore, when the hip has already progressed to obvious end-stage structural damage, and its impact on current quality of life and surgical feasibility is more prominent, a hip-first strategy has a strong practical rationale.

### 4.3 Advantages

The most direct clinical value of a hip-first strategy lies in improving hip range of motion and lower-limb positioning. After successful THA, patients are usually able to achieve better sitting, lying, turning, and standing function. In some cases, release of hip flexion contracture may even produce a certain degree of visible “unfolding,” thereby making subsequent spinal correction easier to perform^28^, ^29^, ^30^^,^^35^. This pathway—restoring hip mobility first and then proceeding to the spinal stage—is particularly important in patients with bilateral bony hip ankylosis.

In addition, a hip-first approach may address pain and local functional impairment at an earlier stage, allowing patients to enter second-stage spinal surgery in a better nutritional, rehabilitative, and psychological condition. For patients in whom spinal kyphosis is evident but does not pose an immediate threat to neurologic function, or in whom overall balance remains relatively well compensated in the short term, performing THA first may represent a more acceptable strategy for the patient and one associated with more controllable perioperative risk.^9^^,^^28^^,^^35^

### 4.4 Limitations and technical considerations

The greatest risk of a hip-first strategy is that, although it resolves the immediate positional obstacle, it leaves THA to be performed before the final pelvic environment has been established. Gu and colleagues showed that, in patients with ankylosing spondylitis undergoing THA, functional cup inclination and anteversion were positively correlated with PT and negatively correlated with SS, indicating that sagittal pelvic alignment has a direct effect on the functional behavior of cup positioning.^19^ The review by Sudhan and colleagues likewise emphasized that there is no fixed cup safe zone applicable to all patients undergoing THA, and that functional cup positioning must be evaluated in the context of the spine–pelvis relationship and postural change.^13^

Therefore, if a hip-first strategy is chosen, the surgeon can no longer rely on the conventional empirical angles used in routine primary THA. Instead, cup positioning must be prospectively adjusted on the basis of whole-spine imaging, the current degree of pelvic retroversion, and the anticipated pelvic anteversion that may occur after subsequent PSO. Zhao and colleagues demonstrated that acetabular anteversion can decrease significantly after PSO(18), and Li and colleagues further noted that the lumbar sagittal profile and the pattern of pelvic motion from standing to sitting influence prosthetic stability when PSO is performed after THA(19). This means that a hip-first approach does not simply involve placing THA earlier in the treatment sequence; rather, it requires the surgeon to estimate the final pelvic orientation before spinal correction has been completed and to design cup positioning in a more individualized manner on that basis. Without such forward-looking planning, the short-term convenience of a hip-first strategy may come at the cost of a higher subsequent risk of impingement or dislocation.

## 5 Summary

In summary, a hip-first strategy is more appropriate for patients in whom hip ankylosis and flexion contracture are the main drivers of overall functional impairment, positioning is difficult, and spinal surgery is not feasible at the current stage. Its principal advantage lies in relieving the most immediate obstacles related to positioning and mobility, thereby creating the conditions necessary for subsequent spinal correction. However, its main limitation is that the pelvic environment on which THA is based does not represent the final postoperative state, and adequate prediction must therefore be made regarding pelvic recoil and changes in functional cup position that may result from later spinal correction. Thus, a hip-first strategy does not ignore spine–pelvis biomechanics; rather, it places current surgical feasibility at the forefront while demanding a higher level of comprehensive preoperative planning.

## 6 Discussion

The management of ankylosing spondylitis with severe hip deformity is challenging not merely because both the spine and the hip may require reconstruction, but because these procedures are undertaken within the same spine–pelvis–hip mechanical chain. In such patients, spinal correction can restore sagittal balance and modify pelvic orientation,^11^^,^^12^ whereas severe hip ankylosis and flexion contracture may markedly restrict anesthesia, surgical positioning, exposure, and even the feasibility of spinal osteotomy^21^, ^22^, ^23^, ^24^. Therefore, the central issue is not simply whether pedicle subtraction osteotomy or total hip arthroplasty should be performed first, but how to determine a sequence that appropriately balances long-term biomechanical reconstruction with immediate surgical feasibility.

From a clinical standpoint, neither a spine-first strategy nor a hip-first strategy should be regarded as universally superior. A spine-first strategy is generally more suitable when thoracolumbar kyphosis is the dominant deformity, compensatory pelvic retroversion remains substantial, and safe positioning for spinal correction can still be achieved.^10^^,^^11^^,^^16^^,^^18^ In this setting, correction of the spinal deformity first may bring the pelvis closer to its final postoperative orientation, thereby providing a more reliable biomechanical basis for subsequent acetabular reconstruction. Hu et al.^16^ demonstrated that both acetabular inclination and anteversion decreased after PSO, and that these changes were significantly correlated with changes in pelvic tilt. This finding supports the view that spinal correction can substantially reshape the functional acetabular environment. Likewise, Zhao et al.^17^ reported a significant decrease in acetabular anteversion after PSO in patients who had previously undergone THA, suggesting that cup orientation established before spinal correction may subsequently become functionally altered. Li et al.^18^ further showed that lumbar sagittal profile influences pelvic orientation and pelvic motion during postural changes after PSO, which may in turn affect postoperative stability.

In contrast, a hip-first strategy is often more reasonable when fixed hip ankylosis, severe flexion contracture, or extreme positional limitation has already become the principal obstacle to treatment. In these patients, THA may improve posture, facilitate sitting or supine positioning, and create the practical conditions necessary for later spinal correction. This rationale is particularly relevant in patients with severe bilateral hip ankylosis or a “folded man” deformity, in whom conventional positioning for spinal surgery may be extremely difficult^21^, ^22^, ^23^. However, a hip-first strategy should not be viewed as a purely local solution. Because pelvic orientation may change after subsequent PSO(18, 19), acetabular component positioning during first-stage THA should not rely solely on conventional empirical targets. Instead, cup placement should be planned with anticipation of later changes in pelvic version and functional cup orientation.

Taken together, the available literature supports an individualized rather than formulaic approach to surgical sequencing. At present, most evidence is derived from retrospective case series, small cohort studies, and radiographic analyses. Although these studies are valuable for clarifying biomechanical principles, they remain insufficient to establish a universally applicable algorithm. Moreover, current reports focus predominantly on spinopelvic parameters and acetabular orientation, while relatively less attention has been paid to broader clinical outcomes such as gait recovery, patient-reported outcomes, complications, reoperation, and long-term implant survival. For this reason, sequencing decisions should still rely primarily on comprehensive preoperative assessment rather than rigid protocol-based recommendations.

In our view, the most practical approach is to evaluate three interrelated dimensions. The first is deformity dominance, namely whether the patient's major disability is primarily driven by sagittal spinal imbalance or by fixed hip deformity. The second is surgical feasibility, that is, whether spinal osteotomy or THA can be safely performed under the existing anesthetic and positional constraints. The third is the anticipated final functional environment, specifically whether the first-stage procedure will create, preserve, or complicate the pelvic and hip conditions required for definitive reconstruction. This framework does not replace clinical judgment, but it may help make sequencing decisions more structured, reproducible, and clinically meaningful. Future studies should move beyond the simple binary question of “spine first or hip first” and instead focus on standardized preoperative assessment and outcome-oriented decision pathways. Prospective multicenter investigations incorporating whole-spine standing imaging, functional spinopelvic parameters, hip structural status, and stage-specific outcomes may provide stronger evidence for optimizing surgical planning in this particularly complex patient population.

## 7 Conclusion

In patients with ankylosing spondylitis and severe hip deformity, neither spine-first nor hip-first surgery is universally appropriate. Surgical sequencing should be individualized according to deformity dominance, current surgical feasibility, and the anticipated postoperative spinopelvic environment. A decision-making strategy based on the overall biomechanics of the spine–pelvis–hip complex may provide a more rational basis for staged reconstruction and improve functional outcomes.

## Guardian patients consent

Not applicable.

## Ethical statement

Not applicable. This article is a review and does not involve human participants, human data, or human tissue directly collected by the authors.

## Credit author statement

NZ conceived the study and drafted the manuscript. HS contributed to the literature review and manuscript revision. YX supervised the study and critically revised the manuscript. All authors read and approved the final manuscript.

## Funding statement

This research received no external funding. None of the authors received any funds or has any financial interests to disclose.

## Conflict of interest

The authors declare that they have no competing interests.

## abbreviations

AS: Ankylosing spondylitis
THA: Total hip arthroplasty
PSO: Pedicle subtraction osteotomy
PT: Pelvic tilt
SS: Sacral slope
PI: Pelvic incidence
LL: Lumbar lordosis
SVA: Sagittal vertical axis
ROM: Range of motion
CT: Computed tomography
